# The Application of Optical Coherence Tomography Angiography in Systemic Hypertension: A Meta-Analysis

**DOI:** 10.3389/fmed.2021.778330

**Published:** 2021-11-08

**Authors:** Wilson Tan, Xinwen Yao, Thu-Thao Le, Anna C. S. Tan, Carol Y. Cheung, Calvin Woon Loong Chin, Leopold Schmetterer, Jacqueline Chua

**Affiliations:** ^1^Singapore Eye Research Institute, Singapore National Eye Centre, Singapore, Singapore; ^2^Yong Loo Lin School of Medicine, National University of Singapore and National University Health System, Singapore, Singapore; ^3^School of Chemical and Biomedical Engineering, Nanyang Technological University, Singapore, Singapore; ^4^SERI-NTU Advanced Ocular Engineering (STANCE), Singapore, Singapore; ^5^National Heart Centre Singapore, National Heart Research Institute Singapore, Singapore, Singapore; ^6^Ophthalmology and Visual Sciences Academic Clinical Program, Duke-National University of Singapore Medical School, Singapore, Singapore; ^7^Department of Ophthalmology and Visual Sciences, The Chinese University of Hong Kong, Shatin, Hong Kong SAR, China; ^8^Department of Clinical Pharmacology, Medical University of Vienna, Vienna, Austria; ^9^Center for Medical Physics and Biomedical Engineering, Medical University of Vienna, Vienna, Austria; ^10^Institute of Molecular and Clinical Ophthalmology, Basel, Switzerland

**Keywords:** hypertension, blood pressure, optical coherence tomography angiography, OCTA, biological marker, biomarker, retina

## Abstract

**Objective:** Multiple studies have compared various optical coherence tomography angiography (OCTA) parameters in participants with systemic hypertension vs. controls and have presented discordant findings. We conducted a meta-analysis to pool together data from different studies to generate an overall effect size and find out whether OCTA parameter(s) significantly differed in participants with systemic hypertension as compared to controls.

**Methods:** We conducted a literature search through a search of electronic databases to identify studies before 19 June 2021, which compared OCTA parameters in non-diabetic participants with systemic hypertension vs. controls. If the OCTA parameter had a minimum number of 3 studies that analyzed it, the mean difference between participants with systemic hypertension and controls were analyzed using a random-effects model.

**Results:** We identified 11 eligible studies. At the macula, 9 studies analyzed vessel density at the superficial capillary plexus (SVD), 7 analyzed vessel density at the deep capillary plexus (DVD), and 6 analyzed the area of the superficial foveal avascular zone (FAZ). Participants with systemic hypertension had significantly lower SVD (standardized mean difference [SMD], −0.50 [−0.70, −0.30], P < 0.00001, *I*^2^ = 63%), lower DVD (SMD, −0.38 [−0.64, −0.13], *P* = 0.004, *I*^2^ = 67%) and larger superficial FAZ (SMD, 0.32 [0.04, 0.61], *P* = 0.020, *I*^2^ = 77%).

**Conclusion:** The eyes of people with systemic hypertension have robustly lower superficial and deep vascular densities at the macula when compared to control eyes. Our results suggest that OCTA can provide information about pre-clinical microvascular changes from systemic hypertension.

## Introduction

Systemic hypertension remains the leading contributor to the global burden of disease and global all-cause mortality, leading to 9.4 million deaths and 212 million lost healthy life years (8.5% of the global total) each year (1). In 2015, an estimated 874 million adults had a systolic blood pressure of 140 mmHg or higher globally (2). Systemic hypertension, damages the body's microvasculature, and leads to increased risk of complications known as target end-organ damage (Chua J), which include cerebrovascular accidents, cardiovascular diseases, renal failure, and retinal vascular disease.

**Figure 1:**
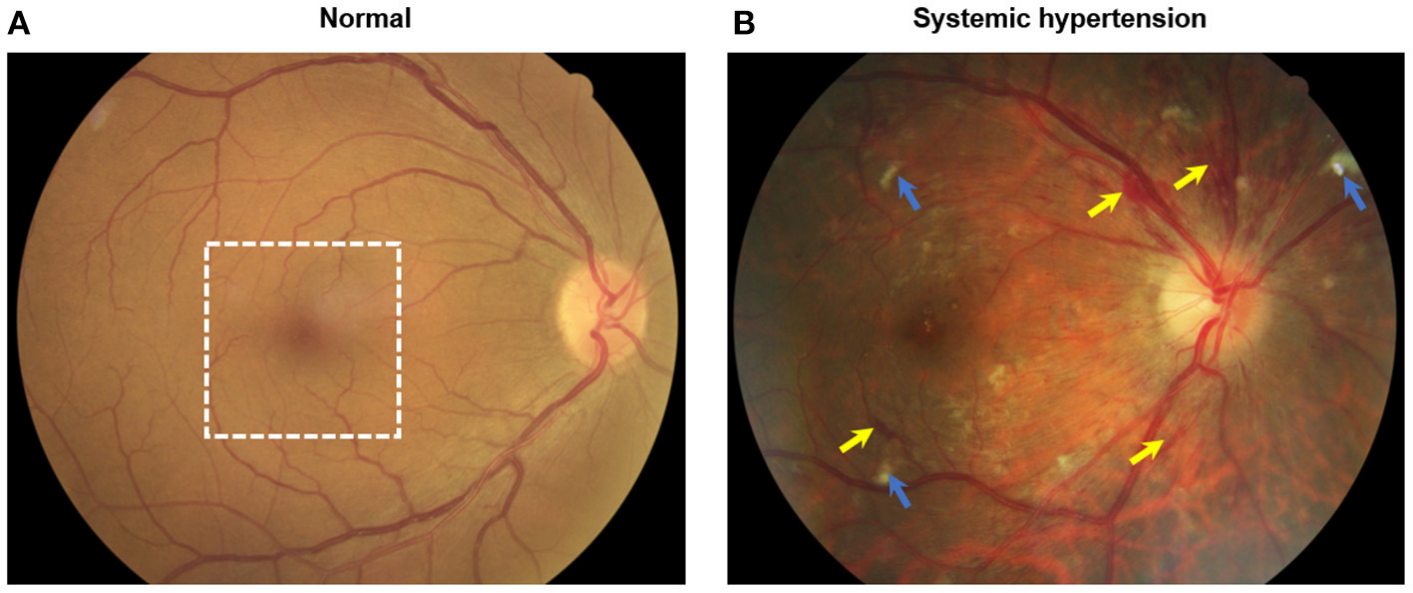
Color fundus photographs of **(A)** an individual without systemic hypertension with normal eye and **(B)** a hypertensive person with signs of moderate hypertensive retinopathy. Notably features include flame hemorrhages (yellow arrows) and cotton wool spots (blue arrows). White dotted box indicates the optical coherence tomography angiography (OCTA, 3 × 3 mm^2^) scanned region as seen in Figure 2.

The retina is a highly vascularized tissue which is susceptible to microvascular damage due to hypertension and retinal imaging provides a unique opportunity to non-invasively assess these pathological changes. The current systems of grading hypertensive retinopathy, the Keith-Wagner-Barker or Wong-Mitchell classification systems, are based on a clinician's subjective assessment of retinal fundus photographs (3). Signs assessed from retinal fundus photographs (e.g., arteriolar narrowing, arteriovenous nicking, hemorrhages) are limited to the visible larger arterioles and venules in the more superficial layers of the retinal circulation and earlier microvascular changes which may be subclinical biomarkers of disease may be missed (Figure 1).

**Figure 2:**
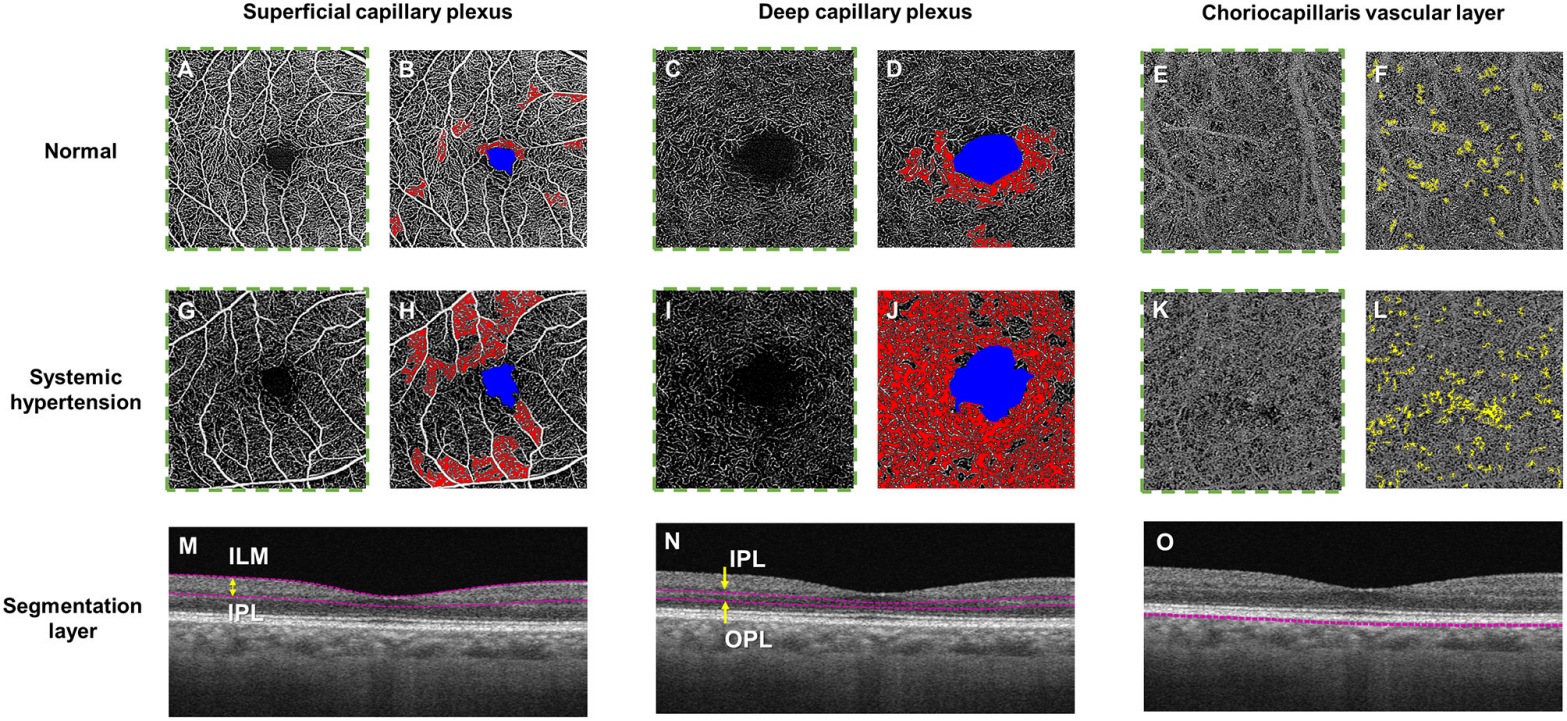
Optical coherence tomography angiography (OCTA) and color-coded maps of a healthy control individual (Top panel; **A–F**) and a hypertensive individual with poorly controlled blood pressure (Middle panel; **G–L**) and the horizontal B-scan images of the corresponding layer (Bottom row, **M–O**). OCTA metrics, including fovea avascular zone (FAZ) area (in blue), retinal vessel density and choriocapillaris flow void deficits, can be calculated automatically. **(M)** The superficial plexus was segmented from the internal limiting membrane (ILM) to the inner plexiform layer (IPL), **(N)** the deep plexus was segmented from the IPL to the outer plexiform layer (OPL) and the choriocapillaris layer was segmented within a thin 10 μm thick slab (31–40 μm) below the retinal pigmented epithelium. The presence of larger non-perfusion area (in red) can be detected in both the superficial and deep capillary plexuses and more choriocapillaris flow voids (in yellow) in a hypertensive eye **(H, J, L)** as compared to the healthy eye **(B, D, F)**.

The advent of optical coherence tomography angiography (OCTA) has provided us with depth-resolved high-resolution images of both the superficial and deep retinal vascular layer and the choroid, without the administration of intravenous dye (4). OCTA technology has the ability to non-invasively assess and quantify the vessels of all layers of the retinal vasculature and choroid (5), in both disease and healthy states (Figure 2). Therefore, detecting pre-clinical changes in the retinal microvascular in response to hypertension, that correlates to disease states in other microvascular systems, may provide the potential opportunity to discover novel objective biomarkers of early microvascular changes. Detecting and monitoring pre-clinical microvascular changes before irreversible end-organ damage occurs may alter practice patterns with earlier and stricter interventions to control blood pressure, with the aim of preventing life-threatening hypertensive-related complications.

Recently, multiple studies have been conducted comparing various quantitative OCTA parameters (e.g., vessel density), in patients with systemic hypertension vs. healthy controls and have presented discordant findings. For example, a 2021 study by Donati et al. (6) found no significant reduction in superficial vessel density in patients with systemic hypertension when compared to healthy controls. However, a 2020 study by Sun et al. (7) found otherwise. We hypothesize that there are quantitative OCTA parameters which significantly differ in patients with systemic hypertension when compared with healthy controls and present a meta-analysis of studies investigating measurements by OCTA in patients with systemic hypertension.

## Methods

This meta-analysis is reported in accordance with the Meta-analysis Of Observational Studies in Epidemiology (MOOSE) group guidelines (8). Minor deviations are described below.

## Search Strategy and Study Selection

We systematically searched PubMed, Embase, The Cochrane Library, Scopus, and Web of Science, for all human studies published until 19 June 2021 in all languages. The concepts identified for this review are (1) OCTA; (2) Hypertension. The keywords searched for the concept OCTA are (1) OCTA; (2) Optical Coherence Tomography Angiography (3) OCT Angiography. The keywords searched for the concept Hypertension are (1) Hypertension; (2) cardiovascular disease; (3) CVD; (4) high blood pressure; (5) high mean arterial pressure; (6) hypertensive crisis; (7) hypertensive crises. The search results were then exported to Endnote where duplicates were excluded. The remaining studies were then screened by Title/Abstract using Rayyan (9) by two independent reviewers (W.T and J.C). Discrepancies between reviewer selections were resolved by the decision of a third independent reviewer (Y.X). Studies selected by Title/Abstract then underwent a full-text review by two independent reviewers (W.T and J.C). Discrepancies between reviewer selections were resolved by the decision of a third independent reviewer (Y.X).

## Inclusion and Exclusion Criteria

Inclusion criteria for studies were: (1) studies with hypertensive patients and non-hypertensive controls; (2) studies which analyzed microvascular parameters using OCTA; (3) cross-sectional or longitudinal studies. We excluded the following studies: (1) reviews; (2) case reports; (3) non-human research; (4) conference presentations or summaries. The inclusion criteria for hypertensive patients were: (1) diagnosis of systemic hypertension as defined by systolic blood pressure ≥140 mmHg, and/or diastolic blood pressure ≥90 mmHg, and/or history of antihypertensive medication, and/or physician diagnosed hypertension in clinic setting. The exclusion criteria for hypertensive patients were: (1) abnormal fundus findings or ophthalmological disease except for hypertensive retinopathy; (2) age <18 years. The inclusion criteria for controls were: (1) no diagnosis of systemic hypertension and the exclusion criteria were: (1) abnormal fundus findings or ophthalmological disease; (2) age <18 years.

## Data Extraction and Synthesis

Studies selected after full-text review underwent data extraction. The data extracted were: (1) title; (2) first author; (3) year of publication; (4) study design; (5) number of hypertensive eyes analyzed; (6) number of hypertensive participants; (7) number of control eyes analyzed; (8) number of control participants; (9) Mean age and sex of hypertensives and controls; (10) Major modifiable cardiovascular risk factor characteristics (10) of hypertensives and controls; (11) OCTA parameters analyzed; (12) mean values of OCTA parameters analyzed; (13) diagnostic criteria; (14) participant selection criteria; (15) method of imaging and analysis used.

One researcher (W.T) performed data extraction, while another researcher (J.C) reviewed the extracted data for possible inclusion in this meta-analysis. Discrepancies between reviewer selections were resolved by the decision of a third independent reviewer (Y.X). As some studies studied both eyes of each participant, analysis was performed at the eye level instead of at the participant level; the total number of eyes is similar to the total number of participants for all meta-analyses as outlined in the results section below. OCTA parameters used for comparison between patients with systemic hypertension and controls were synthesized for analysis if they were reported in a minimum number of 3 papers. The mean and standard deviation of the OCTA parameters from individual studies were combined using a random effects meta-analysis, for both the hypertensive and control group. If a study had the hypertensive patients split into subgroups, the mean and standard deviation of the subgroups were combined to produce a mean and standard deviation for the hypertensive patients as a whole (https://training.cochrane.org/handbook/current/chapter-06; https://www.statstodo.com/CombineMeansSDs_Pgm.php). Similarly, if a study divided a specific OCTA parameter into subgroups, the mean and standard deviation of the subgroups was combined to produce a total mean and standard deviation for the specific OCTA parameter. The difference in mean value of the specific OCTA parameter(s) in patients with systemic hypertension vs. healthy controls was then used as the main outcome measure.

## Assessment of Study Quality

Study quality was assessed with the Newcastle-Ottawa Scale (NOS) for Quality Assessment. Since the NOS is not suitable for cross-sectional studies, adapted scales have been created (11, 12). We adapted the NOS scale for cross-sectional studies (12) for our research question. Studies were graded as unsatisfactory (≤4 stars), satisfactory (5–6 stars), good (7–8 stars) and very good (9–10 stars) (12). One researcher (W.T) performed the quality assessment, while another researcher (J.C) reviewed the assessment. Discrepancies between quality assessment were resolved by the decision of a third independent reviewer (Y.X).

## Statistical Analyses

Meta-analyses of continuous outcomes was conducted with the Cochrane Collaboration's Review Manager software (RevMan 5) (Review Manager (RevMan) [Computer program]. Version 5.4) using an inverse variance with random-effects in the model. We chose random effects instead of fixed effects analysis because of the different OCTA devices used in the studies. The *z*-test was performed to assess the difference between the OCTA measures from the patient cohort and healthy controls, where a *P*-value of <0.05 was considered significant. Statistical heterogeneity of data synthesized was assessed by utilizing the Higgins *I*^2^ value, ranging from 0 to 100%, that measures the degree of inconsistency across studies, and tentatively assigned as low, moderate, and high to *I*^2^ values of 25, 50, and 75% (13).

A potentially important source of heterogeneity is the type of OCTA device used in different studies, as manufacturers' proprietary built-in software algorithms and segmentation algorithms to obtain the specific vascular plexuses vary between machines (Supplementary Figure S1). There was a variety of OCTA machines used in these studies, namely the AngioVue (Optovue Inc., Fremont, CA, USA), Cirrus 5000 AngioPlex (Carl Zeiss Meditec Inc., Dublin, CA., USA), and PLEX Elite 9000 (Carl Zeiss Meditec Inc., Dublin, CA., USA). Both AngioVue and Cirrus AngioPlex 5000 are spectral-domain systems, based on an 800 nm optical window, while PLEX Elite 9000 is a swept-source system that utilizes a longer wavelength (1,060 nm), allowing for better penetration and higher sensitivity at deeper layers such as choroid. In addition, different manufactures employed different techniques to identify blood vessels from the change in the OCT signals induced by the moving blood cells. Both Zeiss AngioPlex and PLEX Elite 9000 employed an optical microangiography (OMAG) algorithm, which calculates the decorrelation in the phase and intensity between repeated B-scans. On the other hand, Optovue AngioVue is based on the split spectrum amplitude decorrelation angiography (SSADA) algorithm, which uses a small sliding window to split the spectrum into small bands and calculates the decorrelation. A small discrepancy exists in layer segmentation provided by each machine as well. Specifically, the segmentation algorithm for the superficial retinal layer slab (which is bounded by the internal limiting membrane and inner plexiform layer) is the same for Zeiss AngioPlex and PLEX Elite 9000 but shifted slightly downward in Optovue by a few microns.

Heterogeneity was investigated using planned subgroup analysis on the different OCTA devices when *I*^2^ was above 50%. We then tested for subgroup differences, considering *p*-values of 0.05 or less as significant. Following Cochrane guidelines (https://training.cochrane.org/handbook/current/chapter-10#section-10-11-4), meta-regression was not performed as there were fewer than 10 studies for each OCTA parameter that was meta-analyzed. To assess publication bias, funnel plots were used.

## Results

Supplementary Figure S2 summarizes the selection process for the 11 eligible studies, using the 2020 Preferred Reporting Items for Systematic Reviews and Meta-Analyses (PRISMA) flow diagram. The literature search yielded 783 studies, of which 374 were unique. Of these, 358 were removed after they were determined to not meet the inclusion/exclusion criteria after a title and abstract screen. The full-text version of the remaining 16 studies relevant to OCTA and hypertension were retrieved and assessed for eligibility. We omitted five studies due to a lack of control group (14, 15), not meeting the diagnostic criteria for systemic hypertension (16), not screening participants for concurrent ophthalmological conditions (17), and involving participants <18 years of age (18).

Table 1 summarizes the key characteristics of the 11 eligible studies that were included for meta-analyses. For the macula, nine studies analyzed the superficial vessel density (6, 7, 19, 20, 22, 24–27), seven analyzed the deep vessel density (6, 7, 19, 20, 22, 24, 25), five analyzed the superficial foveal avascular zone (6, 7, 19, 26, 27), two analyzed vessel density (length) (26, 27), two analyzed skeleton density (19, 22), two analyzed vessel diameter index (19, 22), and one analyzed fractal dimension (19). For the optic nerve head, five studies analyzed peripapillary vessel density (6, 20, 23–25), two analyzed inside disc perfusion density (20, 25), one analyzed vessel caliber (19), and one analyzed vessel density (length) (23). For the choriocapillaris, two studies analyzed density of flow deficits (21, 22), two analyzed size of flow deficits (21, 22), and two analyzed number of flow deficits (21, 22). Nine studies used spectral domain OCTA (SD-OCTA) devices, while two used swept source OCTA (SS-OCTA). Regarding the OCTA models, six studies used the AngioVue (Optovue RTVue XR Avanti; Optovue Inc., Fremont, CA), three studies used the Cirrus 5000 AngioPlex (Carl Zeiss Meditec, Dublin, CA, USA), and two studies used the PLEX Elite 9000 (Carl Zeiss Meditec, Inc., Dublin, USA). All studies were cross-sectional and there were no longitudinal studies published before 19 June 2021. The populations sampled were from China (four), Italy (one), Singapore (two), Germany (one), and South Korea (three). Age of participants was recorded in all studies. Sex of participants was recorded in all studies except for one (22). As for major modifiable cardiovascular risk factors, smoking status was available for three studies (21, 24), BMI was available for one study (24), dyslipidemia status was available for one study (21), diabetic patients were not included in any studies, and mean systolic and diastolic blood pressure readings were available in all studies except for three (19, 22, 26). As for quality assessment, all studies included in our meta-analyses had either a good or very good rating; the complete assessment of study quality is shown in Supplementary Figure S3.

**Table 1:** Key characteristics of the 11 eligible studies.

**References**	**Country**	**Study design**	**Number of hypertensive eyes**	**Number of hypertensives**	**Number of control eyes**	**Number of controls**	**Age and Sex (male)**	**Major modifiable cardiovascular risk factors**	**Type of hypertensive patients**	**Type of OCTA machine used**	**OCTA parameters analyzed**
Xu et al. (19)	China	Cross-sectional	137	77	79	43	**Age:** hypertensives (59.2 +/– 7.6), controls (57 +/– 6.8) **Sex:** hypertensives (26), controls (15)	**Smoking:** – **BMI: –** **Dyslipidemia: –** **Diabetes: –** **Systolic BP: –** **Diastolic BP: –**	77 patients (137 eyes) with untreated hypertension	AngioVue	**Macula:** vessel density, skeleton density, foveal avascular zone, vessel diameter index, fractal dimension **Optic nerve head:** vessel caliber
Hua et al. (20)	China	Cross-sectional	73	73	40	40	**Age:** hypertensives (64.32 +/– 2.99), controls (65.65 +/– 2.89) **Sex:** hypertensives (32), controls (17)	**Smoking: –** **BMI: –** **Dyslipidemia: –** **Diabetes: –** **Systolic BP:** hypertensives (125.76 +/– 12.92), controls (119.6 +/– 8.72) **Diastolic BP:** hypertensives (80.01 +/– 7.88), controls (76.8 +/– 5.92)	**Group A:** 32 hypertensive patients (32 eyes) with intensive BP control (systolic BP < 120) **Group B:** 26 hypertensive patients (26 eyes) with standard BP control (systolic BP 120–140) **Group C:** 15 hypertensive patients (26 eyes) with poor BP control (systolic pressure >140)	AngioVue	**Macula:** vessel density **Optic nerve head:** peripapillary vessel density, inside disc perfusion density
Donati et al. (6)	Italy	Cross-sectional	60	30	30	15	**Age:** hypertensives (54.1 +/– 5.38), controls (52.18 +/– 4.73) **Sex:** hypertensives (15), controls (8)	**Smoking: –** **BMI: –** **Dyslipidemia: –** **Diabetes: –** **Systolic BP:** Hypertensives (130.83 +/– 5.91), Controls (113.2 +/– 7.41) **Diastolic BP:** hypertensives (81.64 +/– 6.09), controls (69.82 +/– 6.73)	**Group 2**: 15 patients (30 eyes) with newly diagnosed hypertension **Group 3**: 15 patients (30 eyes) with treated hypertension	AngioVue	**Macula:** vessel density, foveal avascular zone **Optic nerve head:** peripapillary vessel density
Chua et al. (21)	Singapore	Cross-sectional	116	71	74	41	**Age:** hypertensives (56.75 +/– 9.09), controls (55 +/– 14) **Sex:** hypertensives (44), controls (25)	**Smoking:** hypertensives (2), controls (2) **BMI: –** **Dyslipidemia:** hypertensives (25), controls (11) **Diabetes: –** **Systolic BP:** hypertensives (129.83 +/– 12.46), controls (124 +/– 11) **Diastolic BP:** hypertensives (80.04 +/– 8.36), controls (72 +/– 8)	**Good BP control** (systolic BP < 140 and/or diastolic BP < 90): 53 hypertensive patients (87 eyes) **Poor BP control** (systolic BP > 140 and/or diastolic BP > 90): 18 hypertensive patients (29 eyes)	PLEX Elite 9000	**Choriocapillaris flow deficits (macula):** density, size, number
Terheyden et al. (22)	Germany	Cross-sectional	28	17	31	18	**Age:** hypertensives (56 +/– 19), controls (52 +/– 16) **Sex: –**	**Smoking: –** **Dyslipidemia: –** **Diabetes: –** **Systolic BP: –** **Diastolic BP: –**	17 patients (28 eyes) with hypertensive crisis (systolic BP ≥ 180 and/or diastolic BP ≥ 110)	PLEX Elite 9000	Macula: vessel density, skeleton density, vessel diameter index **Choriocapillaris flow deficits (macula):** density, size, number
Sun et al. (7)	Singapore	Cross-sectional	94	94	46	46	**Age:** Hypertensives (64.77 +/– 9.03), controls (58.3 +/– 4.62) **Sex:** hypertensives (47), controls (21)	**Smoking: –** **BMI: –** **Dyslipidemia: –** **Diabetes: –** **Systolic BP:** hypertensives (150.82 +/– 17.37), controls (123.35 +/– 11.04) **Diastolic BP:** hypertensives (78.64 +/– 8.52), controls (70.97 +/– 6.62)	94 patients (94 eyes) with systemic hypertension	AngioVue	**Macula:** vessel density, foveal avascular zone
Shin et al. (23)	South Korea	Cross-sectional	78	78	90	90	**Age:** hypertensives (61.72 +/– 9.27), controls (60.1 +/– 8.9) **Sex (Male):** hypertensives (37), controls (38)	**Smoking: –** **BMI:** **Dyslipidemia: –** **Diabetes: –** **Systolic BP:** hypertensive: (118.07 +/– 8.56), controls (115.6 +/– 9.7) **Diastolic BP:** hypertensives (81.93 +/– 7.34), controls (79.8 +/– 7.6)	**Group 1**: 38 patients (38 eyes) with hypertension < 10 years **Group 2**: 40 patients (40 eyes) with hypertension ≥ 10 years	Cirrus AngioPlex 5000	**Optic nerve head:** vessel density (length), peripapillary vessel density
Peng et al. (24)	China	Cross-sectional	169	169	30	30	**Age:** hypertensives (53.54 +/– 10.85), controls (53.6 +/– 9.2) **Sex (male):** hypertensives (83), controls (15)	**Smoking:** hypertensives (16), controls (3) **BMI:** hypertensives (24.53 +/– 3.06), controls (24.1 +/– 1.9) **Dyslipidemia: –** **Diabetes: –** **Systolic BP:** hypertensives [with retinopathy (135; range 130–149), no retinopathy (136; range 127–147)], controls (117; range 110–122) **Diastolic BP:** hypertensives [with retinopathy (85; range 80–95), no retinopathy (85; range 79–93)], controls (75; range 65–81)	**Group A:** 113 patients (113 eyes) with hypertensive retinopathy **Group B:** 56 patients (56 eyes) without hypertensive retinopathy	AngioVue	**Macula:** vessel density **Optic Nerve Head:** peripapillary vessel density
Hua et al. (25)	China	Cross-sectional	57	57	40	40	**Age:** Hypertensives (65.67 +/– 3.02), controls (65.65 +/– 2.89) **Sex:** Hypertensives (22), controls (17)	**Smoking: –** **BMI: –** **Dyslipidemia: –** **Diabetes: –** **Systolic BP (mmHg):** hypertensives (120.75 +/– 8.42), controls (119.6 +/– 8.72) **Diastolic BP (mmHg):** hypertensives (78.35 +/– 5.27), controls (76.80 +/– 5.92)	**Group A:** 35 patients (35 eyes) with a history of hypertension for >10 years **Group B:** 22 patients (22 eyes) with a history of hypertension for 5–10 years	AngioVue	**Macula:** vessel density **Optic nerve head:** inside disc perfusion density
Lim (27)	South Korea	Cross-sectional	84	84	117	117	**Age:** hypertensives (58.53 +/– 9.14), controls (56.4 +/– 12.68) **Sex:** hypertensives (38), controls (53)	**Smokers:** hypertensives (24), controls (19) **BMI: –** **Dyslipidemia: –** **Diabetes: –** **Systolic BP:** hypertensives (122.9 +/– 13.8), controls (119.2 +/– 14.4) **Diastolic BP:** hypertensives (82.9 +/– 8.7), controls (81.0 +/– 7.4)	**Group 1:** 32 patients (32 eyes) with hypertension < 5 years **Group 2:** 52 patients (52 eyes) with hypertension ≥ 5 years	Cirrus AngioPlex 5000	**Macula:** vessel density (superficial only), foveal avascular zone
Lee (26)	South Korea	Cross-sectional	85	85	100	100	**Age:** hypertensives (51.8 +/– 12.2), controls (50.4 +/– 12.6) **Sex:** hypertensives (45), controls (51)	**Smoking: –** **BMI: –** **Dyslipidemia: –** **Diabetes: –** **Systolic BP: –** **Diastolic BP: –**	**Group A:** 45 patients (45 eyes) with hypertension of at least 10 years **Group B:** 40 patients (40 eyes) with relieved hypertensive retinopathy (grade IV, <1 year prior)	Cirrus AngioPlex 5000	**Macula:** vessel density (superficial only), foveal avascular zone

*Age is shown as mean +/– SD in years, sex is shown as number of males, smoking is shown as number of smokers, BMI is shown as mean +/– SD in kg/m2, dyslipidemia is shown as number of participants with dyslipidemia, diabetes is shown as number of participants with diabetes, systolic and diastolic BP is shown as mean +/– SD in mmHg*.

## Superficial Vascular Density (SVD) at Macula

**Figure 3:**
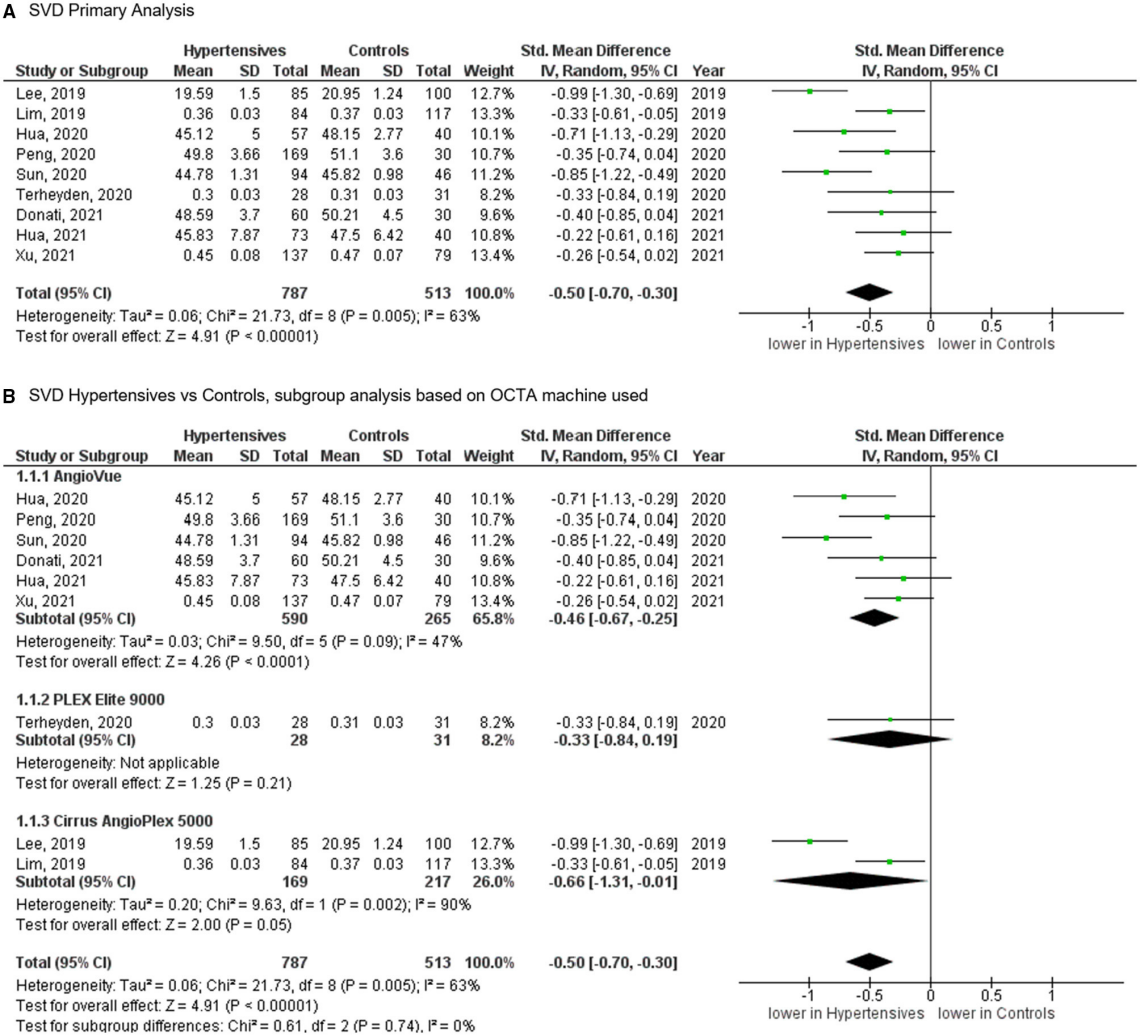
**(A)** Meta-analysis of superficial vascular density (SVD; SMD) and **(B)** SVD subgroup analysis based on OCTA devices for patients with systemic hypertension vs. controls. Mean and standard deviation (SD) are included, with 95% confidence intervals (CIs), heterogeneity scores, and overall effect in an inverse variance (IV) random effects model. The green square size represents the weight attributed to each study based on relative sample size.

Of the 11 studies, nine published results featuring the SVD in 787 eyes of 659 patients with systemic hypertension and 513 eyes of 449 healthy controls. Reduction of the SVD of standardized mean difference [SMD], −0.50 [−0.70, −0.30], *P* < 0.00001 occurred in eyes of patients with systemic hypertension as compared with control eyes (Figure 3). Given the heterogeneity in the SVD analysis was considered significant (*I*^2^ = 63% and *P* = 0.005), we then performed a subgroup analysis by OCTA devices. Our analysis did not reveal any statistically significance heterogeneity introduced by the type of OCTA machine (*I*^2^ = 0% and *P* = 0.740).

## Deep Vascular Density (DVD) at Macula

**Figure 4:**
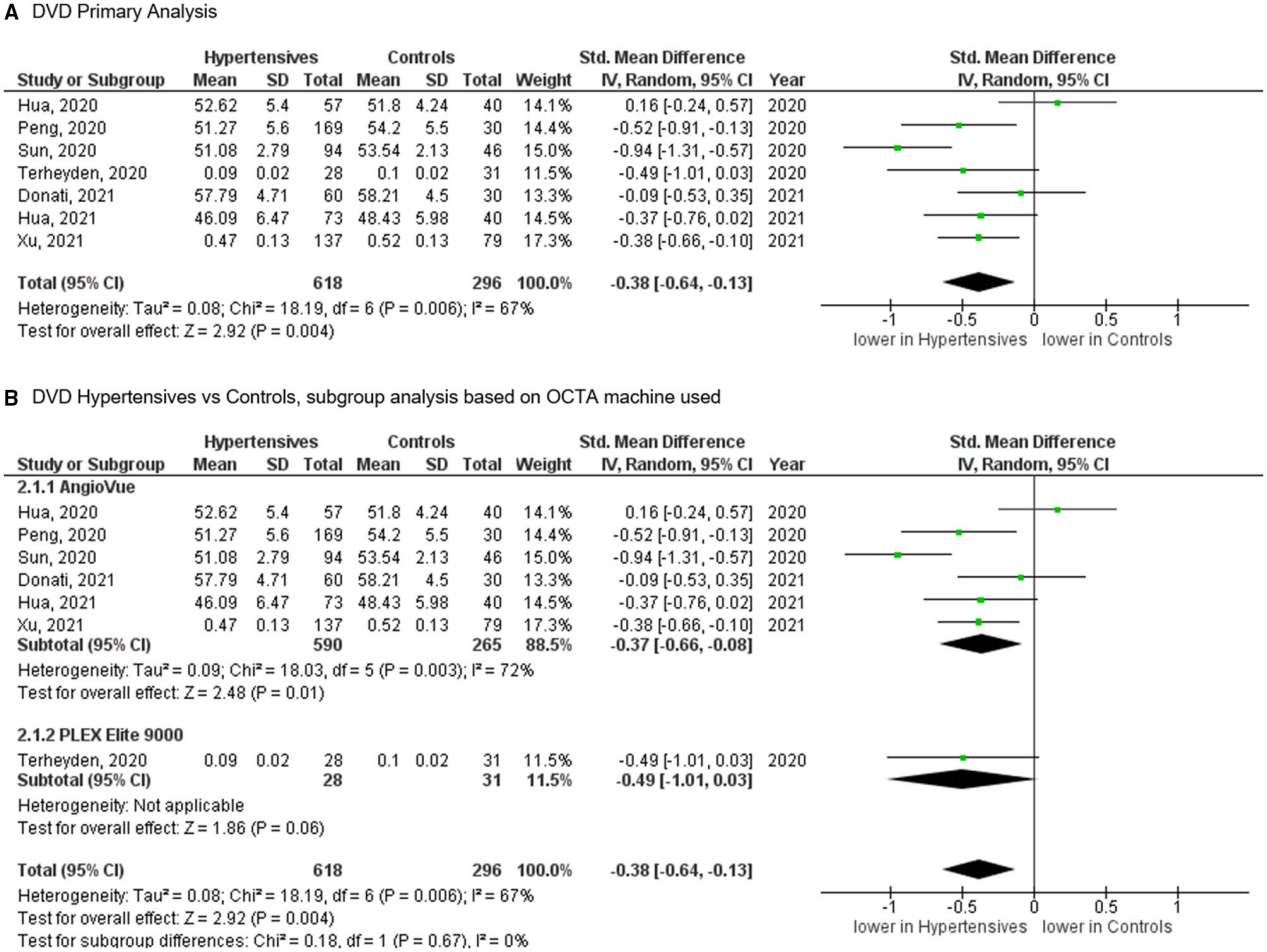
**(A)** Meta-analysis of deep vascular density (DVD; SMD) and **(B)** DVD subgroup analysis based on OCTA devices for patients with systemic hypertension vs. controls. Mean and standard deviation (SD) are included, with 95% confidence intervals (CIs), heterogeneity scores, and overall effect in an inverse variance (IV) random effects model. The green square size represents the weight attributed to each study based on relative sample size.

For the DVD, the meta-analysis of seven studies (Figure 4), totaling 618 eyes of 517 patients with systemic hypertension and 296 eyes of 232 healthy controls, revealed significantly reduced vascular density in patients with systemic hypertension when compared to controls (SMD, −0.38 [−0.64, −0.13], *P* = 0.004). The heterogeneity in the DVD analysis was considered significant (*I*^2^ = 67% and *P* = 0.006). Subgroup analysis by OCTA machines did not show statistically significant heterogeneity (*I*^2^ = 0% and *P* = 0.670).

## Foveal Avascular Zone (FAZ)

**Figure 5:**
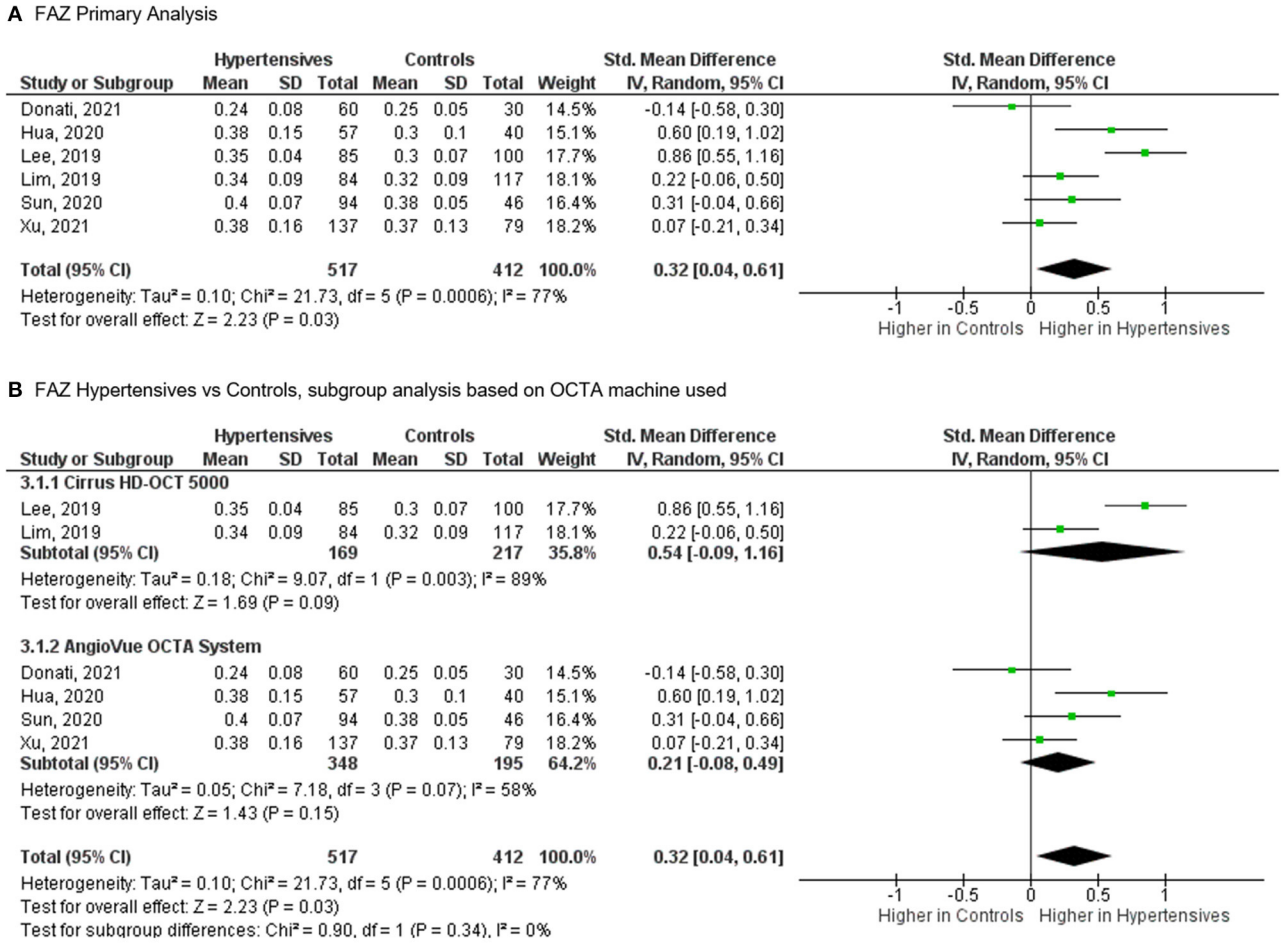
**(A)** Meta-analysis of superficial foveal avascular zone (FAZ; SMD) and **(B)** FAZ subgroup analysis based on OCTA devices for patients with systemic hypertension vs. controls. Mean and standard deviation (SD) are included, with 95% confidence intervals (CIs), heterogeneity scores, and overall effect in an inverse variance (IV) random effects model. The green square size represents the weight attributed to each study based on relative sample size.

For the FAZ, meta-analysis of six studies (Figure 5), totaling 517 eyes of 400 patients with systemic hypertension and 412 eyes of 361 healthy controls, revealed a statistically significant increase in foveal avascularity in patients with systemic hypertension when compared to controls (SMD, 0.32 [0.04, 0.61], *P* = 0.030). The heterogeneity in the FAZ analysis was considered significant (*I*^2^ = 77% and *P* < 0.001). The test for subgroup differences revealed no statistically significant heterogeneity introduced by the type of OCTA machine used to measure the FAZ (*I*^2^ = 0% and *P* = 0.340).

## Peripapillary Vascular Density (PVD)

For PVD, meta-analysis of five studies (Supplementary Figure S4), totaling 407 eyes of 377 patients with systemic hypertension and 204 eyes of 189 healthy controls, revealed no significant difference in vascular density in patients with systemic hypertension when compared to controls (SMD, −0.35 [−1.11, 0.42], *P* = 0.370).

## Additional Analyses

Publication biases were investigated by plotting funnel plots, which revealed a symmetrical distribution of studies about the SMD of −0.50, −0.38, and 0.33, indicating little to no publication bias nor small study bias in the analysis for SVD, DVD and FAZ area, respectively (Supplementary Figures S5–S7).

We also performed a sensitivity analysis by removing the 2020 study by Terheyden et al. (22) as the study involved hypertensive patients with recent history (<7 days prior) of hypertensive crisis as defined by systolic blood pressure greater or ≥180 mmHg and/or diastolic blood pressure ≥120 mmHg. Results remained similar after sensitivity analysis, where both SVD and DVD were significantly reduced in hypertensives as compared to controls (SMD, −0.52 [−0.73, −0.30], *P* < 0.00001, *I*^2^ = 67%) and SMD, −0.37 [−0.66, −0.08], *P* = 0.010, *I*^2^ = 72%; Supplementary Figures S8, S9), respectively.

## Discussion

In this meta-analysis, the data (6, 7, 19–27) shows that systemic hypertension is associated with reduction of vascular density in the superficial capillary plexus (SVD and FAZ area) and deep capillary plexus (DVD) at the macula (Figure 2). This outcome suggests the potential of OCTA technology to serve as tool for pre-clinical retinal microvascular changes in systemic hypertension, where changes in the small retinal vessels may potentially be imaging biomarkers to risk stratify hypertensive complications in end-organs such as the brain, heart, and kidney (28).

## Potential OCTA Measures of Microcirculation in Systemic Hypertension

### Superficial and Deep Vascular Densities

The reduced superficial and deep vascular densities on OCTA that we observed in the hypertensive subjects could be the result of either capillary dropout (structural absence of capillaries) or functional non-perfusion. Since OCTA relies on the change between consecutive scans, it will detect flow only above a minimum threshold (29) and regions that have flow below the slowest detectable flow would therefore be visualized as non-perfusion using the OCTA imaging technique. Coupling OCTA and adaptive optics may be an exciting avenue. Adaptive optics is another novel ocular imaging approach that provides *in vivo* ultra-high-resolution imaging of retinal vessel morphology in humans (30).

### FAZ Area

We observed a weak association between hypertension and FAZ area, which may be explained by the substantial heterogeneity among the studies. Investigations into potential sources revealed that the type of OCTA machine was not the source of the heterogeneity for FAZ area. Previous studies have shown that the size of the FAZ is notoriously variable among normal individuals (31). Another limitation is the difference in reporting of FAZ where traditional analyses (26, 27) (prior to 2020) splits the reporting of superficial vs. deep FAZ, whereas the newer analyses give a combined FAZ parameter. Taken together, it prevents us from recommending the FAZ area as a parameter in systemic hypertension research.

### Peripapillary Vascular Density

We are not able to draw a conclusion on the utility of peripapillary vascular density in differentiating systemic hypertension cases from controls. The wide degree of imprecision of this estimate may be due to the marked inter-individual variation in the blood supply of the optic nerve head, requiring a larger sample size (32).

### Additional OCTA Parameters

In the present meta-analysis, only four OCTA parameters have been meta-analyzed. Other parameters identified through this systematic review may be promising but do not yet have enough data to pool together. These include fractal dimension of the retina, and capillary density inside the optic nerve head (20, 24). Of particular interest is the choroidal circulation. In a 2019 study by Lee et al. (33), choroidal blood was found to increase and be affected before retinal circulation in patients with elevated blood pressure. This may indicate that the choroidal circulation is the earliest vasculature in the eye to be affected in systemic hypertension. However, our systematic review only found two papers, 2021 study by Chua et al. (21) and 2020 study by Terheyden et al. (22), to have compared the patterns of choriocapillaris flow deficits at the macula of patients with systemic hypertension with healthy controls.

## Recommendations for Future Study Designs

Ocular microvascular dysfunction can be due to eye diseases or hypertension. This explains why most studies screened in the meta-analysis excluded participants for concurrent ophthalmological conditions, except for one study (17). As we know that hypertension is a risk factor for age-related macular degeneration (34) and glaucoma (35), an unanswered question now is to determine if hypertensive capillary damage participates to the disease or if it is another mechanism. Future studies wanting to corroborate the level of hypertensive retinopathy/choroidopathy, and the degree of retinal damage could consider including hypertensive patients in control groups.

Apart from eye diseases, three other factors can bias the relationship between hypertension and OCTA: aging, diabetes, and methods of blood pressure measurement. Aging is associated with decreased retinal tissue perfusion (36, 37). OCTA metrics, such as areas of retinal non-perfusion have been found in eyes of patients with diabetes without retinopathy (38, 39). Methods of blood pressure measurement i.e., ambulatory vs. office, can affect the accuracy of blood pressure readings (40). It will be important for future studies to consider confounders during the interpretation of results as confounding variables can distort the observed association between OCTA and hypertension.

It may also be interesting for future studies to note the presence of major modifiable cardiovascular risk factors (10) of study participants apart from hypertension, namely smoking status, body mass index, dyslipidemia, and investigate whether variations in OCTA parameters are influenced by these other factors. This may also allow future meta-analyses to perform meta-regression analyses of the effects of continuous variables such as body mass index, blood pressure, or lipid levels on retinal/choroidal vessel density.

## Potential Use of OCTA as a Quick and Non-Invasive Measure of Microcirculation

### OCTA Parameters and Blood Pressure Control

Several papers have examined how the retinal microcirculation, as measured by OCTA parameters, is affected by blood pressure control. However, pooling these studies is challenging due to differences in stratification of blood pressure control. For example, the 2021 study by Hua et al. (20) attempted to classify hypertensive patients into three groups based on blood pressure control, in accordance with the Systolic Blood Pressure Intervention Trial (SPRINT study) (41), where intensive blood pressure control was defined as systolic blood pressure <120 mmHg; standard blood pressure control was defined as systolic blood pressure 120–140 mmHg; and poor blood pressure control was defined as systolic blood pressure >140 mmHg. On the other hand, the 2021 study by Chua et al. (21) attempted to stratify patients into two groups by defining well-controlled ambulatory blood pressure defined as systolic blood pressure <140 mmHg and/or diastolic blood pressure <90 mmHg and poorly controlled blood pressure defined as systolic blood pressure ≥140 mmHg and/or diastolic blood pressure ≥90 mmHg. The extreme heterogeneity in which patients are currently being stratified in studies make it challenging for meta-analyses to be done. There is a need to harmonize these criteria for future OCTA studies.

### OCTA Parameters and Class of Anti-hypertensive Treatments

It has been proposed that different classes of anti-hypertensive treatments have differing effects at the microvascular levels (42). The hypothesis is, however, largely unproven because of the lack of microvascular imaging. As such, OCTA is an attractive technique to study the effects anti-hypertensive treatments on the microvasculature. However, caution must be taken. For example, though the 2020 study by Peng et al. (24) attempted to investigate the use of anti-hypertensive treatments on OCTA parameters, it was difficult to ascertain the duration the patient was on the specific anti-hypertensive or whether he/she had been recently switched over from a different class. This may erroneously attribute a certain OCTA outcome to a specific class of anti-hypertensive drugs when in fact it was due to a previous medication that the patient was recently on. Considering this, it may be most appropriate for future studies to conduct randomized-control trials where OCTA parameters of untreated/newly diagnosed essential hypertensives starting on different hypertensive medications are measured at various time points longitudinally.

### OCTA Parameters and Hypertensive End-Organ Damage

Whether the OCTA adds value to risk prediction of hypertensive end-organ damage remains to be determined. Two small studies have shown the association of retinal capillary rarefaction and impaired kidney function (estimated glomerular filtration rate levels) in hypertensive individuals (14, 43). To investigate the prognostic value of OCTA parameters for hypertensives end-organ damage, prospective longitudinal follow-up studies can be done to, for example, correlate SVD or DVD values with the incidence/risk of renal failure at 5 years. Correlation of retinal findings with other systemic measures of microvasculature in other organs will help shed further light on the significance of these retinal microvascular changes.

## Conclusion

Our results suggest that certain OCTA parameters can provide objective information about pre-clinical microvascular changes from systemic hypertension and have the potential to act as novel biomarkers of these changes.

## Data Availability Statement

The original contributions presented in the study are included in the article/Supplementary Material, further inquiries can be directed to the corresponding author/s.

## Author Contributions

WT, XY, and JC contributed to the conception and design of the study, literature search, data extraction and synthesis, and assessment of study quality. WT performed the statistical analysis and wrote the first draft of the manuscript. XY and JC wrote sections of the manuscript. All authors contributed to manuscript revision, read, and approved the submitted version.

## Funding

Funding received for this work from the Duke-NUS Medical School (Duke-NUS-KP(Coll)/2018/0009A), the National Medical Research Council (CG/C010A/2017; OFIRG/0048/2017; OFLCG/004c/2018; TA/MOH-000249-00/2018 and MOH-OFIRG20nov-0014), National Research Foundation Singapore (NRF2019-THE002-0006 and NRF-CRP24-2020-0001), A^*^STAR (A20H4b0141), the Singapore Eye Research Institute & Nanyang Technological University (SERI-NTU Advanced Ocular Engineering (STANCE) Program), and the SERI-Lee Foundation (LF1019-1) Singapore.

## Conflict of Interest

The authors declare that the research was conducted in the absence of any commercial or financial relationships that could be construed as a potential conflict of interest.

## Publisher's Note

All claims expressed in this article are solely those of the authors and do not necessarily represent those of their affiliated organizations, or those of the publisher, the editors and the reviewers. Any product that may be evaluated in this article, or claim that may be made by its manufacturer, is not guaranteed or endorsed by the publisher.
