# Supplementary material for: The Application of Optical Coherence Tomography Angiography in Systemic Hypertension: A Meta-Analysis
Source: Front Med (Lausanne). 2021 Nov 8;8:778330. doi: 10.3389/fmed.2021.778330 (PMC8630630; doi:10.3389/fmed.2021.778330)
Supplement: Supplementary file 1 [file Data_Sheet_1.pdf]

Supplementary Material

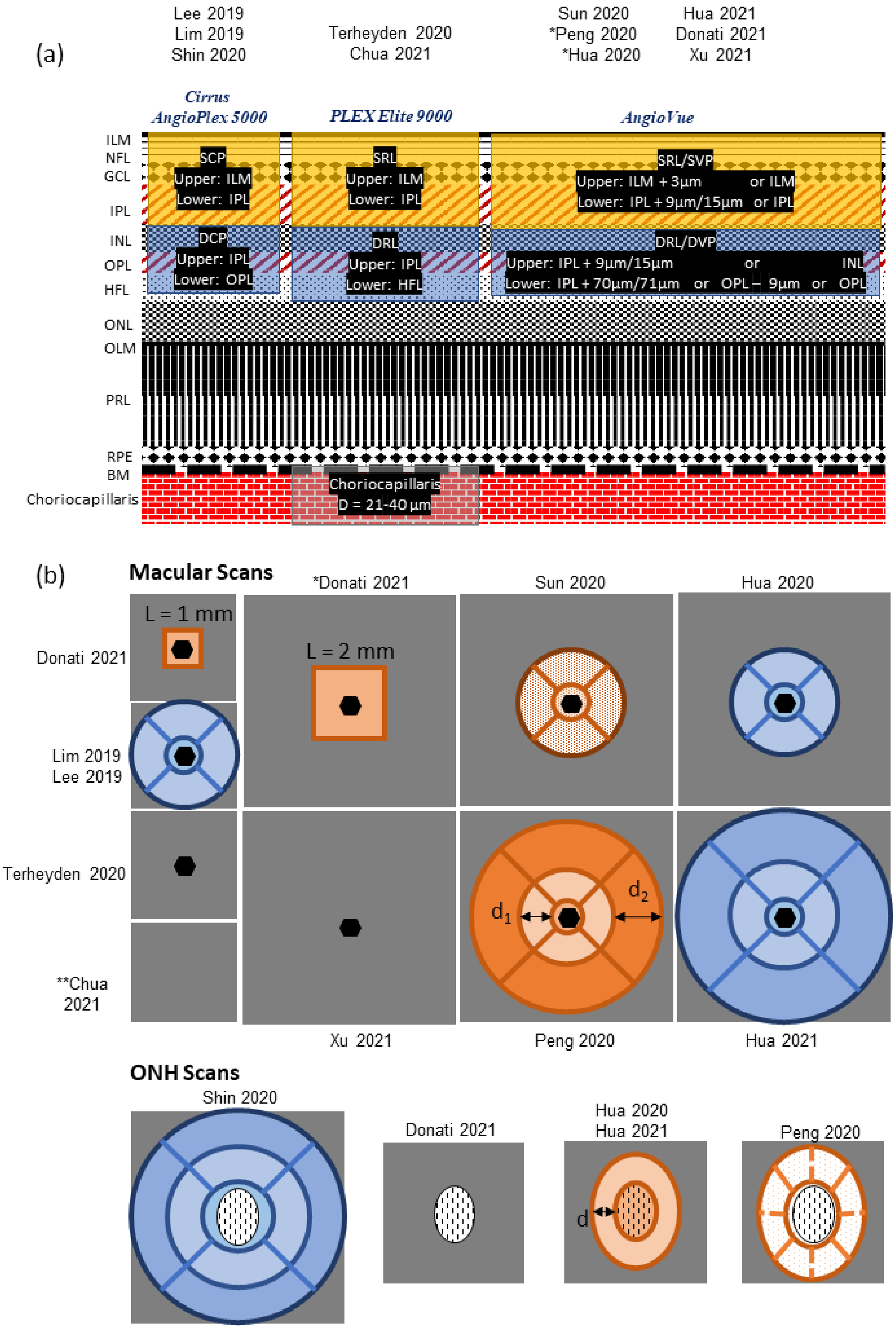
(a)
Lee 2019
Lim 2019
Shin 2020
Terheyden 2020
Chua 2021
Sun 2020
*Peng 2020
*Hua 2020
Hua 2021
Donati 2021
Xu 2021
Cirrus AngioPlex 5000
PLEX Elite 9000
AngioVue
ILM
NFL
GCL
IPL
INL
OPL
HFL
ONL
OLM
PRL
RPE
BM
Choriocapillaris
SCP
Upper: ILM
Lower: IPL
SRL
Upper: ILM
Lower: IPL
SRL/SVP
Upper: ILM + 3µm or ILM
Lower: IPL + 9µm/15µm or IPL
DCP
Upper: IPL
Lower: OPL
DRL
Upper: IPL
Lower: HFL
DRL/DVP
Upper: IPL + 9µm/15µm or INL
Lower: IPL + 70µm/71µm or OPL – 9µm or OPL
Choriocapillaris
D = 21-40 µm
(b)
Macular Scans
*Donati 2021
Sun 2020
Hua 2020
L = 1 mm
L = 2 mm
Donati 2021
Lim 2019
Lee 2019
Terheyden 2020
**Chua 2021
d_1
d_2
Xu 2021
Peng 2020
Hua 2021
ONH Scans
Shin 2020
Donati 2021
Hua 2020
Hua 2021
Peng 2020
d

Supplemental Figure S1: (a) Illustration of layer definitions from three OCT-A machines employed in the selected literature. BM: Bruch's membrane; DCP, deep capillary plexus; DRL, deep retinal layer; DVP, deep vascular plexus; GCL, ganglion cell layer; HFL: Henle's fiber layer; ILM: inner limiting membrane; INL, inner nuclear layer; IPL, inner plexiform layer; NFL, nerve fiber layer; OLM, outer limiting membrane; ONL, outer nuclear layer; OPL, outer plexiform layer; PRL, photoreceptor layers; RPE, retinal pigment epithelium; SCP, superficial capillary plexus; SRL, superficial retinal layer; SVP, superficial vascular plexus; (b) Macular and optic nerve head (ONH) scan patterns and the respective regions of interest (ROIs) adopted for OCTA parameter analysis from the selected studies. Blue circles: ETDRS sub fields (1mm – 3mm or 1mm – 3mm – 6mm concentric circles); Gray square: OCTA image size (3mm x 3mm and 6mm x 6mm macular scans; 6mm x 6mm and 4.5mm x 4.5mm optic disc scans); Solid hexagon: foveal avascular zone (FAZ); Dotted circles: ROI sizes not specified explicitly in the paper; Dashed ellipse: ONH; Orange circles and ellipses: non-standard sub fields with sizes defined in paper. In Peng2020 macular scan, d1 = 1mm, d2 = 1.5mm, with the inner circle conformal to FAZ; In Hua2020 and Hua2021 ONH scan, d = 0.75 mm, with the inner ellipse encircling the ONH. *Donati 2021[1] analyzed two types of macular OCTA images (3mmx3mm and 6mmx6mm) **Chua 2021[2] only did analysis on choriocapillaris.

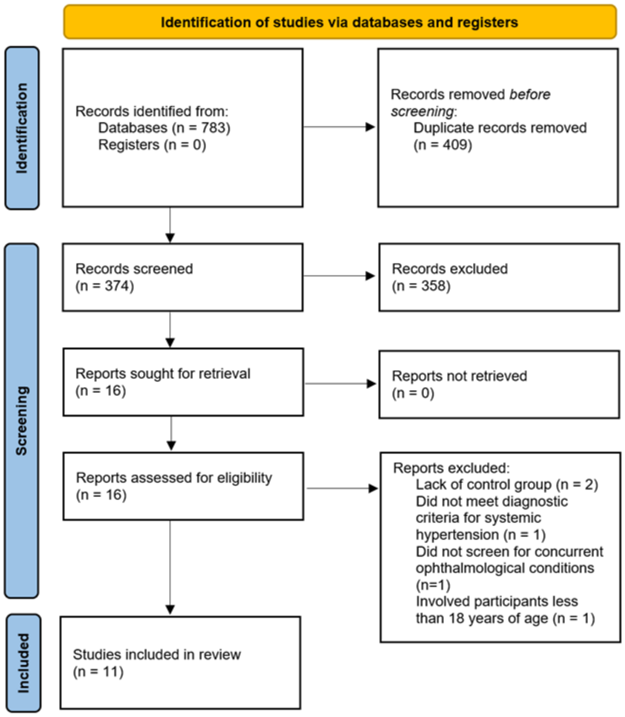

Supplemental Figure S2: Study Selection

Study	Selection (max 5*)				Comparability (max 2*)	Outcome (max 3*)		Total Score
	Representativeness	Selection of Control group	Ascertainment of Disease	Non-Responders	Different Outcome groups comparable	Assessment of Outcome	Statistical Test	
Lee, 2019	*	-	**	-	**	**	*	8
Lim, 2019	*	*	**	-	**	**	*	9
Hua, 2020	*	*	**	-	**	**	*	9
Peng, 2020	*	*	**	-	**	**	*	9
Shin, 2020	*	*	**	-	**	**	*	9
Sun, 2020	*	*	**	-	**	**	*	9
Terheyden, 2020	-	*	*	-	**	**	*	7
Chua, 2021	*	-	**	-	**	**	*	8
Donati, 2021	*	-	**	-	*	**	*	7
Hua, 2021	*	*	**	-	**	**	*	9
Xu, 2021	*	-	**	-	*	**	*	7

Supplemental Figure S3: Study quality assessment using Newcastle-Ottawa Scale, adapted for quality assessment of cross-sectional studies for the meta-analysis “The application of optical coherence tomography angiography in systemic hypertension: A meta-analysis”. Very good studies: 9-10 points, Good studies: 7-8 points, Satisfactory studies: 5-6 points, Unsatisfactory studies: 0-4 points

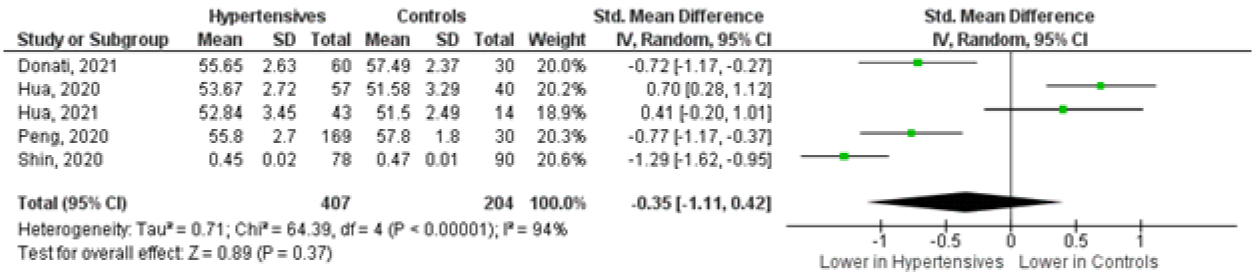

Study or Subgroup	Hypertensives Mean	SD	Total	Controls Mean	SD	Total	Weight	Std. Mean Difference IV, Random, 95% CI
Donati, 2021	55.65	2.63	60	57.49	2.37	30	20.0%	-0.72 [-1.17, -0.27]
Hua, 2020	53.67	2.72	57	51.58	3.29	40	20.2%	0.70 [0.28, 1.12]
Hua, 2021	52.84	3.45	43	51.5	2.49	14	18.9%	0.41 [-0.20, 1.01]
Peng, 2020	55.8	2.7	169	57.8	1.8	30	20.3%	-0.77 [-1.17, -0.37]
Shin, 2020	0.45	0.02	78	0.47	0.01	90	20.6%	-1.29 [-1.62, -0.95]
**Total (95% CI)**			**407**			**204**	**100.0%**	**-0.35 [-1.11, 0.42]**

Heterogeneity: Tau² = 0.71; Chi² = 64.39, df = 4 (P < 0.00001); I² = 94%
Test for overall effect: Z = 0.89 (P = 0.37)

Supplemental Figure S4: Meta-analysis of peripapillary vascular density (PVD; SMD) for patients with systemic hypertension versus controls. Mean and standard deviation (SD) are included, with 95% confidence intervals (CIs), heterogeneity scores, and overall effect in an inverse variance (IV) random effects model. The green square size represents the weight attributed to each study based on relative sample size.

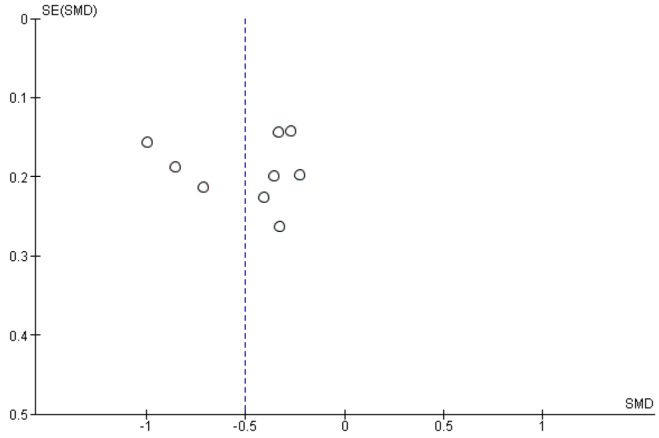

Supplemental Figure S5: Funnel plot for superficial vascular density (SVD) primary analysis

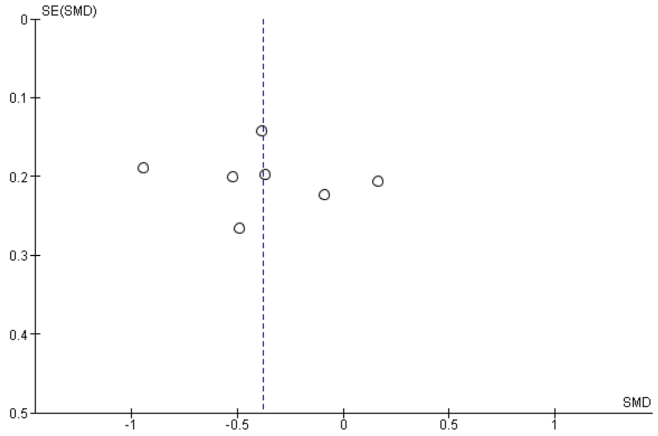

Supplemental Figure S6: Funnel plot for deep vascular density (DVD) primary analysis

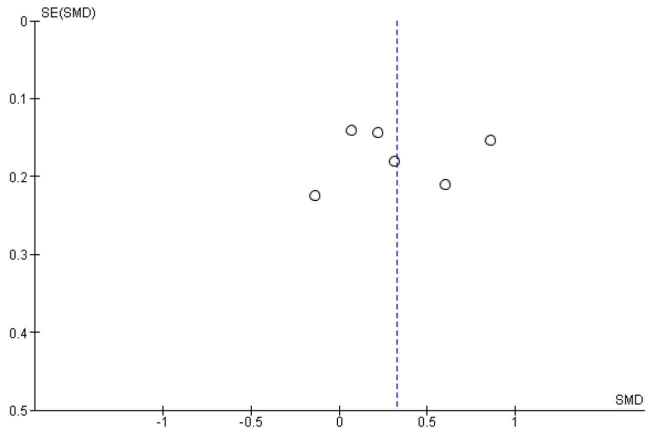

Supplemental Figure S7: Funnel plot for foveal avascular zone (FAZ) primary analysis

	Hypertensives			Controls				Std. Mean Difference
**Study or Subgroup**	**Mean**	**SD**	**Total**	**Mean**	**SD**	**Total**	**Weight**	**IV, Random, 95% CI**
Donati, 2021	48.59	3.7	60	50.21	4.5	30	10.6%	-0.40 [-0.85, 0.04]
Hua, 2020	45.12	5	57	48.15	2.77	40	11.1%	-0.71 [-1.13, -0.29]
Hua, 2021	45.83	7.87	73	47.5	6.42	40	11.8%	-0.22 [-0.61, 0.16]
Lee, 2019	19.59	1.5	85	20.95	1.24	100	13.7%	-0.99 [-1.30, -0.69]
Lim, 2019	0.36	0.03	84	0.37	0.03	117	14.3%	-0.33 [-0.61, -0.05]
Peng, 2020	49.8	3.66	169	51.1	3.6	30	11.7%	-0.35 [-0.74, 0.04]
Sun, 2020	44.78	1.31	94	45.82	0.98	46	12.3%	-0.85 [-1.22, -0.49]
Xu, 2021	0.45	0.08	137	0.47	0.07	79	14.4%	-0.26 [-0.54, 0.02]
**Total (95% CI)**			**759**			**482**	**100.0%**	**-0.52 [-0.73, -0.30]**

Heterogeneity: Tau² = 0.06; Chi² = 21.29, df = 7 (P = 0.003); I² = 67%
Test for overall effect: Z = 4.68 (P < 0.00001)

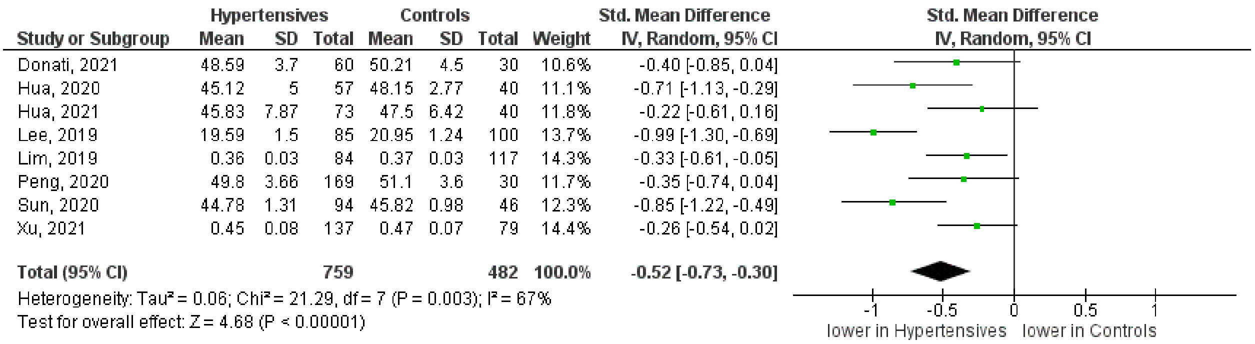

Supplemental Figure S8: SVD sensitivity analysis

	Hypertensives			Controls				Std. Mean Difference
**Study or Subgroup**	**Mean**	**SD**	**Total**	**Mean**	**SD**	**Total**	**Weight**	**IV, Random, 95% CI**
Donati, 2021	57.79	4.71	60	58.21	4.5	30	15.2%	-0.09 [-0.53, 0.35]
Hua, 2020	52.62	5.4	57	51.8	4.24	40	16.0%	0.16 [-0.24, 0.57]
Hua, 2021	46.09	6.47	73	48.43	5.98	40	16.4%	-0.37 [-0.76, 0.02]
Peng, 2020	51.27	5.6	169	54.2	5.5	30	16.3%	-0.52 [-0.91, -0.13]
Sun, 2020	51.08	2.79	94	53.54	2.13	46	16.9%	-0.94 [-1.31, -0.57]
Xu, 2021	0.47	0.13	137	0.52	0.13	79	19.2%	-0.38 [-0.66, -0.10]
**Total (95% CI)**			**590**			**265**	**100.0%**	**-0.37 [-0.66, -0.08]**

Heterogeneity: Tau² = 0.09; Chi² = 18.03, df = 5 (P = 0.003); I² = 72%
Test for overall effect: Z = 2.48 (P = 0.01)

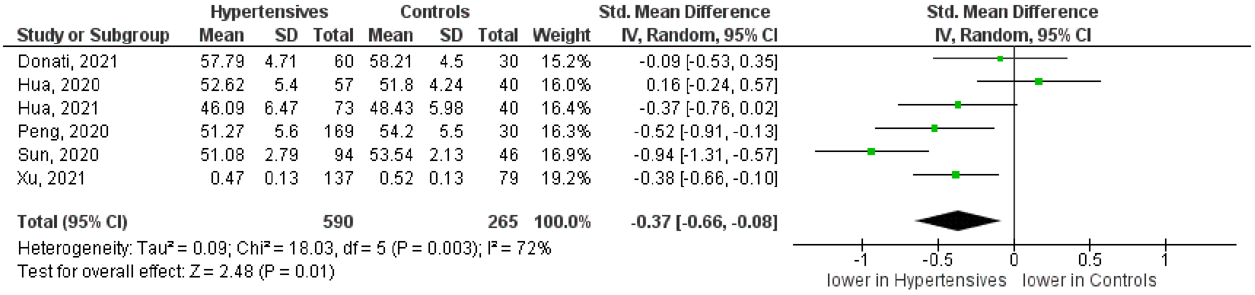

Supplemental Figure S9: DVD sensitivity analysis

References

1. Donati S, Maresca AM, Cattaneo J, Grossi A, Mazzola M, Caprani SM, Premoli L, Docchio F, Rizzoni D, Guasti L, Azzolini C. Optical coherence tomography angiography and arterial hypertension: A role in identifying subclinical microvascular damage? *European Journal of Ophthalmology*. 2021;31:158-165
2. Chua J, Le TT, Tan B, Ke M, Li C, Wong DWK, Tan ACS, Lamoureux E, Wong TY, Chin CWL, Schmetterer L. Choriocapillaris microvasculature dysfunction in systemic hypertension. *Scientific reports*. 2021;11:4603
